# Health and self-perceived barriers to internet use among older migrants: a population-based study

**DOI:** 10.1186/s12889-022-12874-x

**Published:** 2022-03-23

**Authors:** Anne Kouvonen, Teemu Kemppainen, Sakari Taipale, Antero Olakivi, Sirpa Wrede, Laura Kemppainen

**Affiliations:** 1 Faculty of Social Sciences, University of Helsinki, PO Box 54, 00014 Helsinki, Finland; 2 Centre for Public Health, Queen’s University Belfast, Institute of Clinical Science, Block A, Royal Victoria Hospital, Belfast, BT12 6BA UK; 3 Department of Geosciences and Geography, University of Helsinki, PO Box 64, 00014 Helsinki, Finland; 4 Centre Maurice Halbwachs (CNRS/EHESS/ENS), École Normale Supérieure 48, boulevard Jourdan, 75014 Paris, France; 5 Department of Social Sciences and Philosophy, University of Jyvaskyla, PO Box 35, 40014 Jyvaskyla, Finland; 6 Faculty of Social Sciences, University of Ljubljana, Kardeljeva ploščad 5, 1000 Ljubljana, Slovenia; 7 Swedish School of Social Science, University of Helsinki, PO Box 16, 00014 Helsinki, Finland

**Keywords:** Digital information technology, Internet use, Older adults, Migrants, Depression, Barriers to internet use, Cognitive functioning, Chronic conditions

## Abstract

**Background:**

In older adults, including those with a migrant background, ill health is associated with less internet use. However, it is not known what are the specific self-perceived barriers to internet use among older migrants with different health conditions. The aim of this study was to investigate the associations between different health conditions and self-perceived barriers to internet use among older migrants.

**Methods:**

We used the Care, Health and Ageing of Russian-speaking Minority in Finland (CHARM) study, which is a nationally representative survey of community-dwelling Russian-speaking adults aged ≥50 years living in Finland (*N*=1082, 57% men, mean age 63.2 years, standard deviation 8.4 years, response rate 36%). Postal survey data were collected in 2019. Health indicators were self-rated health (SRH), depressive symptoms, cognitive functioning, and doctor-diagnosed conditions. Linear regression analyses were used to investigate the associations between health indicators and a summary scale consisting of the following barriers of internet use: (1) internet use is too complicated and hard to learn; (2) having concerns about safety issues; (3) internet use is too expensive; (4) physical limitations hinder the internet use; (5) memory problems hinder the internet use. In addition, the two most commonly reported barriers (the first two) were examined separately using logistic regression analyses. The analyses were adjusted for age, sex, education, marital status, local language proficiency, and income support, and the health conditions, and were performed with weights accounting for the survey design and non-response.

**Results:**

After adjustments, spine/back problems (*b*=0.13; *p*=0.049), depressive symptoms (*b*=0.40; *p*=0.007), and problems in learning new things (*b*=0.60; *p*<0.0005) were associated with higher level of overall barriers to internet use. In addition, a number of health conditions were associated with individual barriers, albeit some health conditions appeared protective.

**Conclusions:**

In general, older migrants with declining health experience more barriers to internet use than their counterparts with better health. To provide better access to healthcare for older adults, including older migrants, rapidly changing devices, software and apps need to be modified and adapted for those with specific health-related needs.

## Background

In Northern Europe, internet use has become a part of the everyday life for the vast majority of older adults. According to the Statistics Finland survey conducted in 2020, 97% of the 55 to 64-year-olds had used the internet in the last three months. The corresponding figures for 65 to 74-year-olds and 75 to 89-year-olds were 88% and 51%, respectively [1]. In technologically advanced countries with such high connection rates, internet use has practically become a necessity for social inclusion [2]. Public services and everyday communication have increasingly gone online, which means that those who do not use the internet can become excluded not only from the essential health and other public services, but also from social networks, and the society at large. Non-users increasingly consist of the most vulnerable segments of the population: the oldest, the least educated, those with ill health, and those who are socially most isolated [3]. In addition, some studies have shown that ethnic minorities and migrants use the internet less [4–13]. In contemporary societies, digital and social exclusion are deeply intertwined. According to the double jeopardy hypothesis, older migrants are at a particular risk of social exclusion because of their age and migration background [14]. Older migrants with a low socio-economic status and health problems may be especially vulnerable: the intersecting domains of old age, ill health, low socio-economic status, and a migrant background have been shown to increase the risk of social exclusion [15, 16]. At the same time, in transnational context, and amplified by the COVID-19 pandemic, the ability to remain connected with the friends and family has become very strongly dependent on using digital technologies [17].

When populations are ageing, health-related factors are becoming increasingly relevant in understanding the “the digital divide” [18], as deteriorating health conditions can hamper the use of digital technologies. In older adults, various indicators of ill health and disability, such as suboptimal self-rated health (SRH) [19–31], mental ill health [23, 32], chronic conditions [33], frailty [34], declining physical performance [20], limitations in physical capacity [35], vision impairment [35], and cognitive decline, memory limitations and dementia [17, 20, 22, 35–40] have been associated with a lower likelihood of digital technology use. In research, older people are often considered as a homogenous group, even though this group and their reasons for not using the internet are increasingly diverse [41]. Moreover, even though the numbers and proportions of migrants, including older migrants, are rapidly increasing, in the health literature the migration-related aspects of the digital divide are underexplored [8], and little is known about the position of older migrants in relation to digital divide [42]. Most of the studies on health and internet use in older adults have concentrated on majority populations. It is not known what specific barriers to internet use older migrants with different health conditions experience. It would be important to investigate, why those who do not use the internet, do not engage [43]. Furthermore, different health conditions may affect internet use [20], and barriers to use differently. In a Taiwanese study published in 2017, among those aged 50 and over, the most common reasons for non-use were difficulties related to learning, which included factors such as feeling no need, feeling unable or too busy to learn, not interested, and not able to use English [44]. In a Swedish study from 2020 the most common reasons of being a non-user of the internet included not knowing how to use the internet and having no need/interest; whereas no possibility for broadband, being too busy, high costs and security issues were mentioned less often [22]. In a Scottish qualitative study (2017), lack of instructions and guidance, lack of knowledge and confidence, health-related factors and costs emerged as barriers to interacting with tablet computers [45]. In addition, among older migrants, local language proficiency has been found to be the most important barrier to the effective utilisation of the internet for seeking health information [42].

As a Nordic welfare state, Finland has comprehensive social rights guaranteed by legislation, and an extensive tax-financed public health and social welfare sector. Over the last decade, the Finnish state has implemented vast digitalisation projects in the public sector. However, little attention has been paid to the barriers that digitalisation of health and social welfare services may create to the fulfilment of social rights in general, and for people at risk of social exclusion (e.g. older migrants), in particular.

The aim of this study was to investigate the associations between different dimensions of ill health and self-perceived barriers to internet use among older Russian-speaking migrants living in Finland.

## Methods

### Data

The data were drawn from the Care, Health and Ageing of Russian-Speaking Minority in Finland (CHARM) survey that was collected in 2019 [23]. The target population of the study was Russian-speaking community-dwelling older adults (50 years of age or older) who permanently reside in Finland. Russian-speakers are the largest migrant group in Finland, making up about 20% of the country’s migrant population. The study was designed to collect data on participants’ health and well-being, public service experiences, digital inclusion, and access to different types of care. A random sample of 3000 people was drawn from the register of the Digital and Population Data Services Agency; their register covers all persons registered as permanently living in Finland. The sample was stratified by gender. Response rate was 36% (N=1082; 57% men; mean age 63.2 years, standard deviation 8.4 years). The questionnaire was available in Russian and Finnish. Non-response bias was adjusted for by weighting the survey responses. For that, the Finnish Tax Administration register data from 2017 were used to model the response propensity [46]. The tax administration data included information on earnings, capital income, unemployment benefits, and pensions.

Study participation was voluntary, and the participants were informed of their right to withdraw at any time without any consequences. Informed consent was obtained from all participants. The Ethical Review Board in the Humanities and Social and Behavioural Sciences at the University of Helsinki approved the study protocol (#6/2019).

### Health indicators

All study variables were self-reported. The participants were asked if they had ever been diagnosed with any of the following conditions commonly experienced in this age group (yes vs no): high blood pressure / hypertension, spine deterioration, sciatica or other back problem, osteoarthritis or joint deterioration, rheumatoid arthritis or other rheumatoid disease, depression or other mental health problem, asthma, diabetes, memory disorder (Alzheimer’s or other dementia), cancer, some other chronic disease or health problem.

Depressive symptoms were measured by the eight-item Center for Epidemiologic Studies Depression Scale (CES-D )[47], with a score of nine or more points indicating depressive symptoms [48].

Self-rated health (SRH) was assessed with the following question: “In general, would you say your health now is…?” )[49]. Participants rated their health on a 5-point scale. A dichotomous variable was created by categorising the answers into good (good or rather good) and suboptimal (average, fairly poor and poor) [50, 51].

Three aspects of cognitive functioning, that is, self-rated memory, concentration, and learning ability were measured by three items assessing the functioning of 1) memory; 2) concentration; 3) capability to learn new information and things; using a five-point scale (1=very poorly, 2=poorly, 3=satisfactorily, 4=well, 5=very well) [52]. Cognitive functioning variables were dichotomised using 2 as a cut-off point (very poor or poor functioning vs other).

### Self-reported barriers to internet use

Self-reported barriers to internet use were measured with the following question: “Sometimes, the use of the internet can feel difficult. Are there some things that hamper your internet use? (choose 1-3 most relevant options)”. Participants had to choose the relevant options of the following: 1) Internet use is too complicated or hard to learn; (2) I have concerns about internet safety issues; (3) Someone else takes care of the matters that require internet use for me (“proxy use”); (4) Internet use is too expensive; (5) Internet use is difficult because of my poor eyesight or other physical reasons (e.g. stiffness of fingers); (6) Memory problems hinder my internet use; 7) I am not interested in the contents of the internet; 8) I do not have time for internet use; 9) I do not have these kinds of problems with the internet use.

Multiple correspondence analysis (MCA) was applied as a dimension reduction method to create a variable indicating overall barriers to internet use based on items 1, 2, 4, 5, 6 and 9. These six items were selected to indicate the problems of internet use relevant for the perspective of our study. Items 3 (“proxy use”) and 7 (not interested) are not actual barriers to internet use. MCA can be considered analogous to principal component analysis with the possibility of using categorical variables. Similarly to other dimensions reduction techniques, it provides the advantage of increased reliability and content validity in the operationalisation of complex concepts [53]. We performed MCA using Burt’s approach and extracted standard normalized coordinates [54–56]. In addition to MCA approach, the two most common barriers (the first two) were examined separately as outcomes. Thus, we utilise four outcome variables in our analysis.

### Covariates

Covariates included the following socio-demographic characteristics: sex, age (squared), marital status (married or cohabiting vs other), educational level in the country of origin, proficiency in local languages, and receipt of means-tested income support benefit (yes vs no) during the last 12 months as a measure of poverty [57]. The highest educational level in the country of origin was categorised based on the old Soviet educational system as having no or only basic education; vocational training; higher education. Self-reported local language proficiency was assessed on a four-point scale, which for the analysis was dichotomised into good (“I use Finnish or Swedish language in various ways in different situations” and “I can participate on everyday conversations in Finnish or Swedish”), and poor (“I can cope with simple everyday situations in Finnish or Swedish” and “I do not speak either language at all”).

### Statistical analysis

Two nested models were specified to explore the socio-demographic and health-related predictors of self-reported barriers of internet use. Model 1 included all socio-demographic variables, while Model 2 further adjusted for all health indicators. The models of MCA-based outcome variable were estimated as linear regression models, whereas those of single-item outcomes were specified as logistic regression models. Possible curvilinearity regarding the age variable was tested for all outcomes adding age squared to the models. All models were estimated accounting for response-propensity adjusted sampling weights. Moreover, cluster-robust variance estimation was used with administrative regions as clusters. To detect possible multicollinearity, variance inflation factors were examined: the maximum was 1.53, which is well below 10, the oft-used limit of concern. Stata version 15.1 was used for all analyses.

## Results

Table 1 presents the characteristics of the sample and shows that the majority of the participants (75%) were married or cohabiting, 50% had acquired a higher education degree in their country of origin, and 38% rated their local language (Finnish or Swedish) skills as good. As many as 42% of the participants had received means-tested income support over the last 12 months; this indicates widespread poverty in this group as in 2020 the corresponding figure for the whole population of Finland was 7.5%, and for people over 65 years of age it was only 1.8% [58]. Depressive symptoms were commonly experienced (18%). Regarding self-rated health, 60% of the participants rated their health as poor or fairly poor. Self-rated problems with memory, concentration or learning were rare. The prevalence of diagnosed diseases ranged from commonly reported high blood pressure / hypertension (48%) to rarely reported memory disorders (2%). About half of the participants reported that they did not have problems with the internet use. Eleven per cent found internet too complicated or hard to learn, and almost one quarter were worried about internet safety issues. Cost, poor eyesight or other physical problems or memory problems were only rarely perceived as barriers to internet use.

Table 1 further shows bivariate associations between the predictors and outcome variables. Various socio-demographic factors were associated with the three outcome measures. For example, age was positively correlated with the MCA measure of barriers (*r*=0.30; *p*<0.0005) and was associated with a higher likelihood of the experience that internet is too complicated (odds ratio, OR=1.10; *p*<0.0005). Concerning health indicators, there were several statistically significant bivariate associations with the MCA outcome, and the difficulty outcome, but less with the third, the safety outcome. For instance, those who reported that they have a diagnosed memory disorder had a 0.78 units higher mean in the MCA outcome (*p*=0.012) than those without this diagnosis, which equals to 78% of the outcome measure’s standard deviation (SD).

**Table 1:** Characteristics of the sample and the bivariate associations between predictors and outcome variables

			Barriers of internet use (MCA)		Internet too complicated		Internet safety concerns	
Variable	Count	%	Mean	*p*	%	*p*	%	*p*
**Gender**				0.786		0.544		0.941
Female	466	43.1	-0.01		11.59		23.82	
Male	616	56.9	0.01		10.71		23.86	
**Age**	1082	63.2 (8.4)	**0.30**	<0.0005	**1.10**	<0.0005	0.98	0.365
**Marital status**				0.013		<0.0005		0.387
Married or cohabiting	796	74.6	**-0.05**		**9.67**		25.38	
Other	271	25.4	**0.14**		**15.50**		20.66	
**Command of Finnish or Swedish**				<0.0005		<0.0005		0.023
Poor	634	62.2	**0.08**		**14.04**		**21.92**	
Good	385	37.8	**-0.17**		**5.45**		**26.75**	
**Highest education in the country of origin**				<0.0005		<0.0005		0.502
Higher education	541	50.0	**-0.12**		**7.02**		23.66	
Vocational education	459	42.4	**0.09**		**13.94**		24.62	
General/No education/Missing	82	7.6	**0.25**		**21.95**		20.73	
**Income support**				<0.0005		<0.0005		0.020
No	592	58.4	**-0.12**		**7.26**		**27.03**	
Yes	421	41.6	**0.11**		**15.44**		**19.95**	
**Internet problems**								
Use is too complicated and it is too hard to learn	120	11.1	.	.	.	.	.	.
I am concerned about internet safety issues	258	23.8	.	.	.	.	.	.
Someone else than myself takes care of the matters that require internet use	160	14.8	.	.	.	.	.	.
Use of internet is too expensive	38	3.5	.	.	.	.	.	.
Use of internet is complicated by the limited eyesight, hearing or other physical reasons	56	5.2	.	.	.	.	.	.
Memory problems hinder the internet use	20	1.9	.	.	.	.	.	.
I have not faced any of such problems	563	52.0	.	.	.	.	.	.
**Diagnosed diseases**								
High blood pressure, hypertension	515	47.6	**0.13**	<0.0005	**13.79**	<0.0005	24.47	0.703
Spine deterioration, sciatica or other back problem	344	31.8	0.07	0.095	11.52	0.410	26.45	0.102
Osteoarthritis, joint deterioration	304	28.1	**0.14**	0.012	**15.46**	<0.0005	23.03	0.740
Rheumatoid arthritis or other rheumatoid disease	121	11.2	**0.23**	0.027	15.70	0.203	25.62	0.151
Depression or other mental health problems	106	9.8	0.16	0.155	**16.04**	0.008	19.81	0.301
Asthma	72	6.7	0.14	0.144	9.72	0.942	25.00	0.687
Diabetes	144	13.3	0.14	0.226	11.81	0.971	26.39	0.587
Memory disorders (Alzheimer's or dementia)	19	1.8	**0.78**	0.012	**31.58**	0.007	21.05	0.294
Cancer	76	7.0	0.12	0.258	10.53	0.999	18.42	0.857
Other chronic disease or health problem	374	34.6	0.10	0.081	**13.64**	0.020	21.39	0.062
**Depressive symptoms (CES-D)**				<0.0005		0.024		0.009
No	772	71.4	**-0.13**		**9.46**		**22.41**	
Yes	194	17.9	**0.39**		**18.56**		**30.41**	
Missing	116	10.7	**0.21**		**9.48**		**22.41**	
**Self-rated health**				<0.0005		<0.0005		0.055
Good or fairly good	431	40.2	**-0.29**		**5.34**		21.35	
Average to poor	640	59.8	**0.18**		**14.69**		25.47	
**Memory**: poor or very poor	64	6.0	**0.55**	0.006	**28.13**	0.002	17.19	0.410
**Concentration**: poor or very poor	27	2.6	**0.79**	<0.0005	**33.33**	<0.0005	11.11	0.261
**New information and learning**: poor or very poor	79	7.6	**0.82**	<0.0005	**35.44**	<0.0005	24.05	0.976

Footnote:

Statistical tests performed with cluster-robust variance estimation & survey weights

Results with a *p*-value ≤ 0.05 in bold

Cell contents for age: count, mean (SD); correlation, *p*; odds ratio, OR, *p*; OR, *p*

To determine whether health conditions were associated with self-reported overall barriers to internet use after accounting for potential confounders, we regressed self-reported barriers (MCA outcome) on covariates and the presence of different health conditions. As Table 2 shows, barriers of internet use seemed to increase with age (Model 2.1: *b*=0.04; *p*<0.0005; Model 2.2: *b*=0.03; *p*<0.0005); the squared term was not statistically significant and was thus omitted to retain a parsimonious model. Higher education was associated with fewer barriers: Model 2.1 shows a statistically significant gradient where vocational education adds 0.19 units (*p*=0.007, while the lowest educational level adds 0.36 units (*p*=0.025) to the MCA outcome (SD=1.00). With the inclusion of health indicators, these results lost some power, but the pattern was similar. Regarding health indicators (Model 2.2), spine/back problems (*b*=0.13; *p*=0.049), depressive symptoms (*b*=0.40; *p*=0.007), and difficulties in learning new things (*b*=0.60; *p*<0.0005) were statistically significant. In terms of the standard deviation (SD) of the outcome variable, the effect sizes ranged from 13% (spine/back) to 60% (difficulties in learning new things).

**Table 2:** The associations of covariates and health conditions with overall barriers to internet use

	**Model 2.1**				**Model 2.2**			
	*b*	*p*	95% CI		*b*	*p*	95% CI	
**Female**	-0.03	0.561	-0.13	0.07	-0.09	0.164	-0.21	0.04
**Age**	**0.04**	<0.0005	0.02	0.05	**0.03**	<0.0005	0.02	0.04
**Married or cohabiting**	-0.10	0.122	-0.23	0.03	-0.08	0.255	-0.21	0.06
**Good command of Finnish/Swedish**	-0.05	0.448	-0.17	0.08	-0.02	0.743	-0.13	0.09
**Highest education in the country of origin**								
Vocational education	**0.19**	0.007	0.06	0.32	**0.14**	0.040	0.01	0.27
General/No education/Missing	**0.36**	0.025	0.05	0.66	0.32	0.077	-0.04	0.67
**Receipt of income support**	0.02	0.710	-0.11	0.16	-0.07	0.360	-0.21	0.08
**Diagnosed diseases**								
High blood pressure, hypertension	.	.	.	.	0.10	0.086	-0.01	0.21
Spine deterioration, sciatica or other back problem	.	.	.	.	**0.13**	0.049	0.00	0.26
Osteoarthritis, joint deterioration	.	.	.	.	-0.04	0.401	-0.14	0.06
Rheumatoid arthritis or other rheumatoid disease	.	.	.	.	0.03	0.754	-0.19	0.26
Depression or other mental health problems	.	.	.	.	0.08	0.603	-0.23	0.39
Asthma	.	.	.	.	-0.17	0.252	-0.47	0.13
Diabetes	.	.	.	.	-0.03	0.718	-0.22	0.15
Memory disorders (Alzheimer's or dementia)	.	.	.	.	0.40	0.259	-0.32	1.13
Cancer	.	.	.	.	0.01	0.930	-0.31	0.34
Other chronic disease or health problem	.	.	.	.	-0.09	0.320	-0.26	0.09
**Depressive symptoms (CES-D)**								
Yes	.	.	.	.	**0.40**	0.007	0.13	0.67
Missing	.	.	.	.	**0.28**	0.014	0.06	0.49
**Self-rated health**: average to poor	.	.	.	.	0.10	0.175	-0.05	0.25
**Memory**: poor or very poor	.	.	.	.	-0.16	0.223	-0.44	0.11
**Concentration**: poor or very poor	.	.	.	.	-0.10	0.774	-0.80	0.61
**New information and learning**: poor or very poor	.	.	.	.	**0.60**	<0.0005	0.35	0.84
Constant	-2.23	<0.0005	-2.82	-1.64	-2.01	<0.0005	-2.48	-1.54
n	942				908			
R squared	0.106				0.167			

Footnotes:

Cluster-robust variance estimation. Survey weights accounted for

Results with a *p*-value ≤ 0.05 in bold

*CI* confidence interval

Tables 3 and 4 display the associations between the covariates, health conditions and the two most common barriers to internet use. In Models 3.1 and 3.2 (Table 3), age, proficiency of Finnish/Swedish, and education appeared as consistent and statistically significant predictors of feeling that internet is too complicated and hard to learn. Again, with increasing age, this barrier became more common (Model 3.2: odds ratio, OR=1.10; 95% confidence interval (CI): 1.09-1.11); and like above, the square term was not statistically significant. Mastering well the local languages seemed to lessen the risk of experiencing this barrier (Model 3.2: OR=0.62; 95% CI: 0.40-0.95). Finally, there was a clear educational gradient with ORs increasing from 1.73 (vocational education; 95% CI: 1.22-2.46) to 5.36 (lowest level; 95% CI: 1.91-15.05).

**Table 3:** The associations of covariates and health conditions with the experience that internet is too complicated and hard to learn

	Model 3.1				Model 3.2			
	OR	*p*	95% CI		OR	*p*	95% CI	
**Female**	1.27	0.250	0.84	1.92	0.99	0.975	0.60	1.64
**Age**	**1.11**	<0.0005	1.10	1.12	**1.10**	<0.0005	1.09	1.11
**Married or cohabiting**	0.85	0.329	0.62	1.17	0.78	0.196	0.54	1.13
**Good command of Finnish/Swedish**	**0.58**	0.001	0.42	0.80	**0.62**	0.027	0.40	0.95
**Highest education in the country of origin**	.	.	.	.	.	.	.	.
Vocational education	**1.81**	<0.0005	1.44	2.26	**1.73**	0.002	1.22	2.46
General/No education/Missing	**4.04**	0.010	1.39	11.75	**5.36**	0.001	1.91	15.05
**Receipt of income support**	1.87	0.069	0.95	3.69	1.58	0.243	0.73	3.38
**Diagnosed diseases**	.	*.*	.	.	.	.	.	.
High blood pressure, hypertension	.	*.*	.	.	1.44	0.158	0.87	2.41
Spine deterioration, sciatica or other back problem	.	*.*	.	.	0.66	0.105	0.40	1.09
Osteoarthritis, joint deterioration	.	*.*	.	.	**1.53**	0.027	1.05	2.22
Rheumatoid arthritis or other rheumatoid disease	.	*.*	.	.	1.07	0.907	0.34	3.33
Depression or other mental health problems	.	*.*	.	.	2.03	0.132	0.81	5.13
Asthma	.	*.*	.	.	**0.33**	0.025	0.12	0.87
Diabetes	.	*.*	.	.	**0.63**	0.047	0.40	0.99
Memory disorders (Alzheimer's or dementia)	.	*.*	.	.	8.86	0.060	0.91	85.99
Cancer	.	*.*	.	.	0.81	0.663	0.31	2.09
Other chronic disease or health problem	.	*.*	.	.	1.27	0.472	0.67	2.41
**Depressive symptoms (CES-D)**	.	*.*	.	.	.	.	.	.
Yes	.	*.*	.	.	1.93	0.074	0.94	3.98
Missing	.	*.*	.	.	0.50	0.070	0.24	1.06
**Self-rated health**: average to poor	.	*.*	.	.	1.02	0.913	0.70	1.49
**Memory**: poor or very poor	.	*.*	.	.	0.50	0.103	0.21	1.15
**Concentratio**n: poor or very poor	.	*.*	.	.	**0.31**	0.048	0.10	0.99
**New information and learning**: poor or very poor	.	*.*	.	.	**4.53**	<0.0005	2.22	9.24
Constant	0.00	<0.0005	0.00	0.00	0.00	<0.0005	0.00	0.00
n	942				908			
Pseudo R squared	0.187				0.240			

Footnotes:

Cluster-robust variance estimation. Survey weights accounted for

Results with a *p*-value ≤ 0.05 in bold

*OR* odds ratio; *CI* confidence interval

**Table 4:** The associations of covariates and health conditions with having concerns about internet safety

	Model 4.1				Model 4.2			
	OR	*p*	95% CI		OR	p	95% CI	
**Female**	0.95	0.715	0.75	1.22	0.90	0.441	0.68	1.18
**Age**	**1.54**	0.003	1.16	2.05	**1.68**	0.007	1.15	2.46
**Age squared**	**1.00**	0.002	0.99	1.00	**1.00**	0.005	0.99	1.00
**Married or cohabiting**	1.08	0.570	0.83	1.39	1.07	0.649	0.80	1.42
**Good command of Finnish/Swedish**	1.30	0.055	0.99	1.70	**1.35**	0.045	1.01	1.80
**Highest education in the country of origin**								
Vocational education	1.13	0.553	0.75	1.71	1.02	0.931	0.63	1.65
General/No education/Missing	1.14	0.643	0.66	1.97	0.98	0.942	0.54	1.76
**Receipt of income support**	**0.69**	0.009	0.52	0.91	**0.64**	0.004	0.47	0.86
**Diagnosed diseases**								
High blood pressure, hypertension	.	*.*	.	.	1.02	0.893	0.75	1.40
Spine deterioration, sciatica or other back problem	.	*.*	.	.	**1.45**	0.032	1.03	2.04
Osteoarthritis, joint deterioration	.	*.*	.	.	0.81	0.133	0.61	1.07
Rheumatoid arthritis or other rheumatoid disease	.	*.*	.	.	0.98	0.936	0.66	1.46
Depression or other mental health problems	.	*.*	.	.	0.65	0.273	0.30	1.41
Asthma	.	*.*	.	.	0.92	0.809	0.46	1.82
Diabetes	.	*.*	.	.	1.05	0.871	0.56	1.99
Memory disorders (Alzheimer's or dementia)	.	*.*	.	.	2.37	0.486	0.21	26.78
Cancer	.	*.*	.	.	0.82	0.669	0.33	2.04
Other chronic disease or health problem	.	*.*	.	.	**0.67**	0.015	0.48	0.92
**Depressive symptoms (CES-D)**								
Yes	.	*.*	.	.	**2.13**	0.024	1.11	4.11
Missing	.	*.*	.	.	**2.33**	0.003	1.33	4.06
**Self-rated health**: average to poor	.	*.*	.	.	1.31	0.147	0.91	1.89
**Memory**: poor or very poor	.	*.*	.	.	0.84	0.556	0.46	1.51
**Concentration:** poor or very poor	.	*.*	.	.	0.30	0.220	0.04	2.07
**New information and learning**: poor or very poor	.	*.*	.	.	**1.86**	0.043	1.02	3.40
Constant	0.00	<0.0005	0.00	0.00	0.00	0.006	0.00	0.01
n	942				908			
Pseudo R squared	0.021				0.056			

Footnotes:

Cluster-robust variance estimation. Survey weights accounted for

Results with a *p*-value ≤ 0.05 in bold

*OR* odds ratio; *CI* confidence interval

Regarding health indicators, having osteoarthritis (OR=1.53; 95% CI: 1.05-2.22 and experienced difficulties in learning new things (OR=4.53; 95% CI: 2.22-9.24) were associated with a higher likelihood of reporting that internet use is too complicated and hard to learn. In contrast, if a participant had asthma (OR=0.33; 95% CI: 0.12-0.87), diabetes (OR=0.63; 95% CI: 0.40-0.99) or problems with concentration (OR=0.31; 95% CI: 0.10-0.99), they were less likely to report this particular barrier to internet use.

**Fig. 1:**
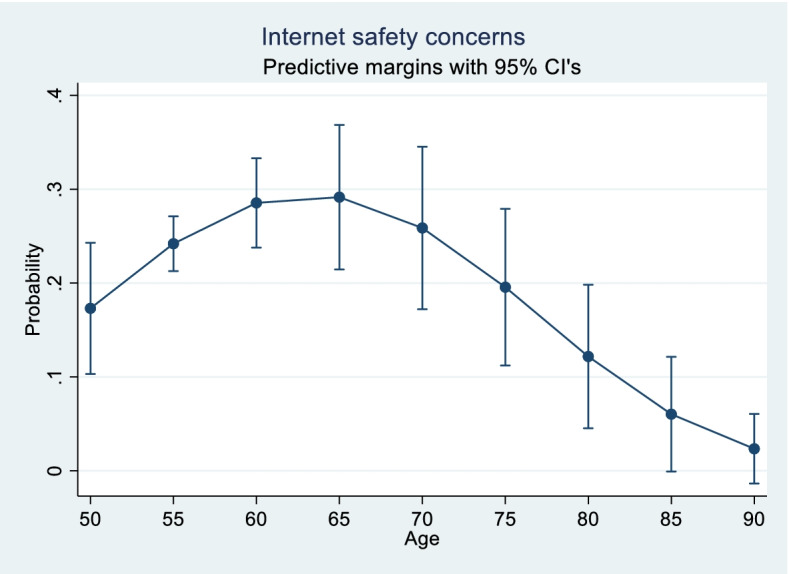
Probability of internet safety concerns as a function of age. Predictive margins from Model 4.2

In terms of experiencing internet related safety concerns, age had a curvilinear pattern with the outcome (Fig. 1; Table 4): as Fig. 1 shows, the probability of such concerns increases to almost thirty percent at 65 years, after which it starts to markedly decrease.

In contrast to the results in Table 3, good command of Finnish/Swedish was associated with a higher likelihood that the participant was concerned about the internet safety (Model 4.2: OR=1.35; 95% CI: 1.01-1.80). Receipt of income support was related to a lower likelihood of such concern (Model 4.2: OR=0.64; 95% CI: 0.47-0.86).

Model 4.2 further shows that back problems (OR=1.45; 95% CI: 1.03-2.04), depressive symptoms (OR=2.13; 95% CI: 1.11-4.11*p*=0.024), and problems with learning new things (OR=1.86; 95% CI: 1.02-3.40) were associated with a higher likelihood of having concerns about internet safety issues, whereas those in the very heterogenous category of “other diseases” were less likely to report this issue (OR=0.67; 95% CI: 0.48-0.92).

We conducted two robustness checks: (1) a model where the depressive symptoms variable did not include a separate category for missing cases; and (2) an estimation without survey weights. Concerning the MCA outcome, the results for the following variables were found to be fully robust: age, depression symptoms, and learning problems. Regarding the experience of internet being too complicated, the following results were fully robust: age, education, asthma, and learning problems. Correspondingly for the last outcome, fully robust results were found for age, income support and depressive symptoms. (Results are available by request.)

## Discussion

Based on a representative survey among older Russian-speaking migrants in Finland, this study investigated the associations between different health conditions and self-reported barriers to internet use.

Our study confirms earlier findings that people do not have just one simple barrier to internet use [43]. The most commonly mentioned barriers were that the internet is too complicated or hard to learn, and safety concerns. However, slightly over half of the participants reported that they did not have any problems with the internet use.

In the present study, depressive symptoms were consistently associated with self-reported barriers to internet use. Earlier studies have shown an association between internet use and depression in older adults [23, 33, 59–61]. The findings from a systematic review indicated a positive association between internet use and good mental health and wellbeing among older adults [32]. The psychosocial links connecting internet use and mental health can include enhanced interpersonal interactions, increased access to resources and services as well as empowered social inclusion [32]. Internet use can decrease loneliness and social isolation [59], which are associated with depression [62]. In particular, internet use for social purposes has been shown to indirectly impact wellbeing via reduced loneliness and increased social engagement, whereas internet use for informational and instrumental (i.e., managing personal administration and daily activities) purposes has been shown to impact well-being through engagement in a wider range of activities [63].

In our study, even though depressive symptoms were associated with more barriers, diagnosed mental disorders were not. It is possible that in a highly digitalised service system, some level of digital inclusion is required to be even able to obtain a diagnosis. On the other hand, since the diagnosis may date several years or even decades back in time, current self-reported depressive symptoms may better indicate the current mental health status of the participant.

In our study, those who reported problems in their cognitive functioning, more specifically in their ability to learn new things, were more likely to experience barriers to internet use. This is in line with several recent studies showing a lower probability of internet use among older people with lower cognitive functioning [17, 39, 40, 64]. A previous US study showed that only 4% of older adults who experienced a new onset dementia between 2011 and 2014, used digital health technology in 2014 [20]. Cognitive decline can make it difficult to use the internet, particularly as it demands constant learning of new skills. On the other hand, internet use and digital literacy may help reduce the risk of cognitive impairment and dementia in midlife and older adults [12, 36, 37, 65, 66], and smartphone apps and internet use have been shown to have potential for the enhancement of cognitive competence of older people [38, 67]. Regular and versatile internet use may directly contribute to the accumulation of cognitive reserve [66]. In light of these results, it would be important to develop such functions, programs and apps that can be more easily used by older adults with reduced cognitive function [40].

In the present study, reporting spine deterioration, sciatica or other back problem was associated with a higher likelihood of reporting overall barriers to internet use. This result is in line with an earlier US study on older adults that showed an association between increased physical capacity impairment and decreased internet use [35].

Compared to older adults belonging to the majority population, older migrants face not only issues related to ageing but also challenges arising from the migration context, including language and cultural barriers as well as physical separation from their families and friends residing in their country of origin or in other countries [68]. This means that older migrants can benefit from the use of the internet in numerous additional ways. The internet can help overcome minor difficulties with the local language, access content in their native language, foster a sense of independence, maintain and strengthen social relationships, and in general enrich their postmigration daily lives [68]. Previous studies have indeed shown that the use of internet not only supports active ageing but also helps older migrants cope with difficulties and losses posed by migration and expand their transnational lifestyles [69].

### Methodological considerations

There are a number of strengths in this study. First, rather than looking at only one general indicator of ill health, we considered a wide range of health conditions. Second, we examined various self-perceived barriers to internet use rather than simply internet non-use. Earlier studies have emphasised the importance of looking at gradations in digital inclusion and exclusion rather than just a simple binary divide [41, 70]. Third, we used a representative sample of older migrants of the largest migrant group in Finland. Fourth, we controlled for a number sociodemographic and socioeconomic factors that can affect both health and barriers to internet use.

However, this study has some limitations. First, our data were observational and cross-sectional and thus do not allow causal inferences. Second, we relied on self-reported measures which can cause recall bias and misclassification that may impact findings.

Third, even though not a direct limitation as such, our study investigated barriers to internet use among older migrants living in a country with an exceptionally high internet penetration, also among older adults [1]. Fourth, our sample included only Russian-speaking long-term migrants, and the participants were relatively highly educated and all were community-dwelling. Fifth, the numbers of those with poor cognitive functioning were low due to the fact the cognitive impairment makes it difficult to participate in surveys. It is possible that also those with other health conditions were underrepresented in the study. It would be important to replicate the study in other countries, other migrant groups and among those living in supportive care settings. A recent Swedish study indicated that the numbers and proportions reported about older people’s internet use are most likely to be greatly overestimated, particularly among the oldest old [22].

Finally, the sample size for some of the examined health conditions was small, reducing the ability to detect statistically significant results.

Despite these limitations, the present findings help advance the current knowledge about experienced barriers to internet use in older migrants.

## Conclusions

In the present study, we found that older migrants with ill health experience more barriers to internet use than those with better health. The inability to use the internet can have very negative ramification for older adults’ health and daily lives as the services and information will be difficult to access without using the internet [71]. Inability to use digital information technology can put older adults at a disadvantage in terms of their ability to live and function independently, perform many essential everyday tasks successfully and effectively, and maintain social relationships [39]. When populations are ageing, health-related factors are becoming increasingly relevant in understanding the “the digital divide”: younger cohorts are more skilled in their internet use compared to older cohorts, but in time every cohort will face ageing and declining health [17]. The rapid pace of technological change means that also future older adults will have problems with accessing and adopting new technologies [72]. In order to further facilitate the adoption of digital technologies among older adults, including older migrants, rapidly changing devices, software and apps need to be modified and adapted for those with specific health-related needs, such as cognitive impairment, sensory issues, declined motor coordination skills or mental health problems. Different language and easy language options should be available. To achieve this goal, it would be important for digital service providers and designers to involve older adults with varying health conditions and from different cultural and socioeconomic backgrounds in hardware, software and app development processes. There is a particular need in these development processes to involve expertise on the situations of older migrants who find the internet use too complicated, have safety concerns, and/or have cognitive impairments, or mental health problems, since these persons face a specific risk of digital exclusion. There is a further need in social policy to ensure that if these people face digital exclusion, their digital exclusion does not lead to a wider exclusion from health and social welfare services and the wider society. In practice, this means that for securing equal access for people who are vulnerable, service providers need to develop practices that can identify people who are at risk, and secure non-digital pathways or socially sustainable support for using digital services.

## Data Availability

The datasets used and analysed during the current study are available from the corresponding author on reasonable request.

## Abbreviations

CES-D: Center for Epidemiologic Studies Depression Scale
CHARM: Care, Health and Ageing of Russian-speaking Minority in Finland study
CI: Confidence interval
OR: Odds ratio
SRH: Self-rated health

## References

[1] Use of the Internet for following the media and for communication has increased. Statistics Finland. 2020. http://www.stat.fi/til/sutivi/2020/sutivi_2020_2020-11-10_tie_001_en.html. Accessed 26 Aug 2021.

[2] van Deursen AJAM, van Dijk JAGM (2019). The first-level digital divide shifts from inequalities in physical access to inequalities in material access. New Media Soc..

[3] Helsper E, Reisdorf B (2017). The emergence of a “digital underclass” in Great Britain and Sweden: changing reasons for digital exclusion. New Media Soc..

[4] Choi NG, Dinitto DM (2013). The digital divide among low-income homebound older adults: Internet use patterns, ehealth literacy, and attitudes toward computer/internet use. J Med Internet Res..

[5] Werner JM, Carlson M, Jordan-Marsh M, Clark F (2011). Predictors of computer use in community-dwelling ethnically diverse older adults. Hum Factors..

[6] Gordon NP, Hornbrook MC (2018). Older adults’ readiness to engage with eHealth patient education and self-care resources: A cross-sectional survey. BMC Health Serv Res..

[7] Nguyen A, Mosadeghi S, Almario C V. Persistent digital divide in access to and use of the Internet as a resource for health information: Results from a California population-based study. Int J Med Inform. 2017;103 March:49–54. 10.1016/j.ijmedinf.2017.04.008 28551001

[8] Samkange-Zeeb F, Borisova L, Padilla B, Bradby H, Phillimore J, Zeeb H (2020). Superdiversity, migration and use of internet-based health information - Results of a cross-sectional survey conducted in 4 European countries. BMC Public Health..

[9] Yoon H, Jang Y, Vaughan PW, Garcia M (2020). Older Adults’ Internet Use for Health Information: Digital Divide by Race/Ethnicity and Socioeconomic Status. J Appl Gerontol..

[10] Mitchell UA, Chebli PG, Ruggiero L, Muramatsu N (2019). The Digital Divide in Health-Related Technology Use: The Significance of Race/Ethnicity. Gerontologist..

[11] Massey P, Langellier B, Sentell T, Manganello J (2017). Nativity and language preference as drivers of health information seeking: examining differences and trends from a U.S. population-based survey. Ethn Heal..

[12] D’Orsi E, Xavier AJ, Rafnsson SB, Steptoe A, Hogervorst E, Orrell M (2018). Is use of the internet in midlife associated with lower dementia incidence? Results from the English Longitudinal Study of Ageing. Aging Ment Health..

[13] Arcury TA, Sandberg JC, Melius KP, Quandt SA, Leng X, Latulipe C (2020). Older Adult Internet Use and eHealth Literacy. J Appl Gerontol..

[14] Blakemore K, Boneham M (1994). Age, race & ethnicity: A comparative approach.

[15] Scharf T, Keating N, Scharf T, Keating N (2012). Conceptualising social inclusion. From Exclusion to Inclusion in Old Age: A Global Challenge.

[16] Torres S, Scharf T, Keating N (2012). International migration: patterns and implications for exlusion in old age. From Exclusion to Inclusion in Old Age: A Global Challenge.

[17] Baldassar L, Wilding R. Migration, Aging, and Digital Kinning: The Role of Distant Care Support Networks in Experiences of Aging Well. Gerontologist. 2020;60:313–21. 10.1093/geront/gnz156 31812983

[18] Ang S, Lim E, Malhotra R (2021). Health-Related Difficulty in Internet Use Among Older Adults: Correlates and Mediation of Its Association With Quality of Life Through Social Support Networks. Gerontologist..

[19] Hong YA, Cho J (2017). Has the digital health divide widened? Trends of health-related internet use among older adults from 2003 to 2011. Journals Gerontol - Ser B Psychol Sci Soc Sci..

[20] Levine DM, Lipsitz SR, Linder JA (2018). Changes in everyday and digital health technology use among seniors in declining health. Journals Gerontol - Ser A Biol Sci Med Sci..

[21] König R, Seifert A, Doh M (2018). Internet use among older Europeans: an analysis based on SHARE data. Univers Access Inf Soc..

[22] Anderberg P, Skär L, Abrahamsson L, Berglund JS (2020). Older people’s use and nonuse of the internet in Sweden. Int J Environ Res Public Health..

[23] Kouvonen A, Kemppainen L, Ketonen EL, Kemppainen T, Olakivi A, Wrede S. Digital information technology use, self-rated health, and depression: population-based analysis of a survey study on older migrants. J Med Internet Res. 2021;23. 10.2196/20988 PMC8240805 34125069

[24] Yoon H, Jang Y, Xie B (2016). Computer use and computer anxiety in older Korean Americans. J Appl Gerontol..

[25] Gracia E, Herrero J (2009). Internet use and self-rated health among older people: A national survey. J Med Internet Res..

[26] Medeiros FL, Xavier AJ, Schneider IJC, Ramos LR, Sigulem D, D’Orsi E (2012). Digital inclusion and functional capacity of older adults living in Florianópolis, Santa Catarina, Brazil (EpiFloripa 2009-2010). Rev Bras Epidemiol..

[27] Burns P, Jones SC, Caputi P, Iverson D (2018). Are older Australians with chronic diseases online?. Heal Promot J Aust..

[28] Yu RP, McCammon RJ, Ellison NB, Langa KM (2016). The relationships that matter: Social network site use and social wellbeing among older adults in the United States of America. Ageing Soc..

[29] Falk Erhag H, Ahlner F, Rydberg Sterner T, Skoog I, Bergström A (2019). Internet use and self-rated health among Swedish 70-year-olds: A cross-sectional study. BMC Geriatr..

[30] Hong YA, Zhou Z, Fang Y, Shi L (2017). The Digital Divide and Health Disparities in China: Evidence From a National Survey and Policy Implications. J Med Internet Res..

[31] Matthews K, Nazroo J, Marshall A (2019). Digital inclusion in later life: Cohort changes in internet use over a ten-year period in England. Ageing Soc..

[32] Forsman AK, Nordmyr J (2017). Psychosocial Links Between Internet Use and Mental Health in Later Life: A Systematic Review of Quantitative and Qualitative Evidence. J Appl Gerontol..

[33] Choi NG, Dinitto DM (2013). Internet use among older adults: Association with health needs, psychological capital, and social capital. J Med Internet Res..

[34] Lee DR, Lo JC, Ramalingam N, Gordon NP (2021). Understanding the Uptake of Digital Technologies for Health-Related Purposes in Frail Older Adults. J Am Geriatr Soc..

[35] Gell NM, Rosenberg DE, Demiris G, LaCroix AZ, Patel KV (2015). Patterns of technology use among older adults with and without disabilities. Gerontologist..

[36] Krug R de R, D’Orsi E, Xavier AJ. Association between use of internet and the cognitive function in older adults, populational longitudinal study EpiFloripa Idoso. Rev Bras Epidemiol. 2019;22:e190012. 10.1590/1980-549720190012 30892475

[37] Xavier AJ, D’orsi E, De Oliveira CM, Orrell M, Demakakos P, Biddulph JP (2014). English longitudinal study of aging: Can internet/e-mail use reduce cognitive decline?. Journals Gerontol - Ser A Biol Sci Med Sci..

[38] Berner J, Rennemark M, Jogréus C, Anderberg P, Sköldunger A, Wahlberg M (2015). Factors influencing Internet usage in older adults (65 years and above) living in rural and urban Sweden. Health Informatics J..

[39] Czaja SJ, Charness N, Fisk AD, Hertzog C, Nair SN, Rogers WA (2006). Factors predicting the use of technology: Findings from the Center for Research and Education on Aging and Technology Enhancement (CREATE). Psychol Aging..

[40] Park S, Kim B (2020). Predictors of internet use among older adults with diabetes in South Korea: Survey study. JMIR Med Informatics..

[41] van Deursen AJAM, Helsper EJ (2015). A nuanced understanding of Internet use and non-use among the elderly. Eur J Commun..

[42] Chen X, Östlund B, Frennert S. Digital Inclusion or Digital Divide for Older Immigrants? A Scoping Review. In: Gao Q, Zhou J, editors. Human Aspects of IT for the Aged Population. Technology and Society. HCII 2020. Lecture Notes in Computer Science. Springer, Cham.; 2020. p. 176–90.

[43] Helsper EJ, Reisdorf BC (2013). A quantitative examination of explanations for reasons for internet nonuse. Cyberpsychol Behav Soc Netw..

[44] Hsu HC (2017). Inequality in Internet Use across Areas and Older Adults in Taiwan. J Healthc Commun..

[45] Vaportzis E, Clausen MG, Gow AJ. Older adults perceptions of technology and barriers to interacting with tablet computers: A focus group study. Front Psychol. 2017;8 OCT:1–11. 10.3389/fpsyg.2017.01687 PMC5649151 29071004

[46] Laaksonen S, Stjernberg M, Vaattovaara M, Kemppainen T, Kortteinen M, Lönnqvist H (2015). Tackling city-regional dynamics in a survey using grid sampling. Surv Res Methods..

[47] Radloff L (1977). The CES-D Scale: A Self-Report Depression Scale for Research in the General Population. Appl Psychol Meas..

[48] Briggs R, Carey D, O’Halloran AM, Kenny RA, Kennelly SP (2018). Validation of the 8-item Centre for Epidemiological Studies Depression Scale in a cohort of community-dwelling older people: data from The Irish Longitudinal Study on Ageing (TILDA). Eur Geriatr Med..

[49] Hays RD, Sherbourne CD, Mazel RM (1993). The RAND 36-Item Health Survey 1.0. Health Econ..

[50] Lyytikäinen L, Kemppainen T (2016). Regional inequalities in self-rated health in Russia: What is the role of social and economic capital?. Soc Sci Med..

[51] Stenholm S, Virtanen M, Pentti J, Oksanen T, Kivimäki M, Vahtera J (2020). Trajectories of self-rated health before and after retirement: Evidence from two cohort studies. Occup Environ Med..

[52] TOIMIA Functioning Measures Database: Self-rated memory, concentration, and learning ability. 2011. https://www.terveysportti.fi/apps/dtk/tmi/article/tmm00047/search/muisti. Accessed 16 Feb 2021.

[53] Kemppainen L, Kemppainen T, Skogberg N, Kuusio H, Koponen P (2018). Immigrants‘ use of health care in their country of origin: the role of social integration, discrimination and the parallel use of health care systems. Scand J Caring Sci..

[54] Greenacre M (2007). Correspondence Analysis in Practice.

[55] Le Roux B, Rouanet H. Multiple Correspondence Analysis. CA: Thousand Oaks: SAGE Publications, Inc; 2010.

[56] Multivariate Statistics Reference Manual. Release 14. Stata Press; 2015.

[57] Kela [Social Insurance Institution]. Social assistance. https://www.kela.fi/web/en/social-assistance. Accessed 26 Aug 2021.

[58] Finnish Institute of Health and Welfare. Social assistance, recipients aged 65 and over, as % of total population of same age. 2020. https://sotkanet.fi/sotkanet/en/taulukko/?indicator=sw5xAQA=&region=s07MBAA=&year=sy5zBAA=&gender=t&abs=f&color=f&buildVersion=3.0-SNAPSHOT&buildTimestamp=202103120740. Accessed 8 Sep 2021.

[59] Cotten SR, Ford G, Ford S, Hale TM (2014). Internet use and depression among retired older adults in the United States: A longitudinal analysis. Journals Gerontol - Ser B Psychol Sci Soc Sci..

[60] Cotten SR, Ford G, Ford S, Hale TM (2012). Internet use and depression among older adults. Comput Human Behav..

[61] Hamer M, Stamatakis E (2014). Prospective study of sedentary behavior, risk of depression, and cognitive impairment. Med Sci Sports Exerc..

[62] Cacioppo J, Hughes M, Waite L, Hawkley L, Thisted R (2006). Loneliness as a specific risk factor for depressive symptoms: cross-sectional and longitudinal analyses. Psychol Ageing..

[63] Szabo A, Allen J, Stephens C, Alpass F (2019). Longitudinal Analysis of the Relationship between Purposes of Internet Use and Well-being among Older Adults. Gerontologist..

[64] Huxhold O, Hees E, Webster NJ (2020). Towards bridging the grey digital divide: changes in internet access and its predictors from 2002 to 2014 in Germany. Eur J Ageing..

[65] Williams BD, Pendleton N, Chandola T (2020). Cognitively stimulating activities and risk of probable dementia or cognitive impairment in the English Longitudinal Study of Ageing. SSM - Popul Heal..

[66] Ihle A, Bavelier D, Maurer J, Oris M, Kliegel M (2020). Internet use in old age predicts smaller cognitive decline only in men. Sci Rep..

[67] Klimova B, Valis M. Smartphone applications can serve as effective cognitive training tools in healthy aging. Front Aging Neurosci. 2018;9 JAN:1–4. 10.3389/fnagi.2017.00436 PMC5770789 29379432

[68] Zhang J. Aging in cyberspace: Internet use and quality of life of older Chinese migrants. J Chinese Sociol. 2016;3.

[69] Khvorostianov N, Elias N, Nimrod G (2012). “Without it I am nothing”: The internet in the lives of older immigrants. New Media Soc..

[70] Livingstone S, Helsper E (2007). Gradations in digital inclusion: children, young people and the digital divide. New Media Soc..

[71] Holthe T, Halvorsrud L, Karterud D, Hoel KA, Lund A (2018). Usability and acceptability of technology for community-dwelling older adults with mild cognitive impairment and dementia: A systematic literature review. Clin Interv Aging..

[72] Chiu CJ, Liu CW. Understanding older adult’s technology adoption and withdrawal for elderly care and education: Mixed method analysis from national survey. J Med Internet Res. 2017;19. 10.2196/jmir.7401 PMC5694030 29101093

